# Two decades of resistance surveillance by the British Society for Antimicrobial Chemotherapy: design, development, delivery, deficiencies and future directions

**DOI:** 10.1093/jac/dkaf247

**Published:** 2025-10-27

**Authors:** Alasdair P MacGowan, Rosy Reynolds, Benjamin J Parcell, David M Livermore

**Affiliations:** Bristol Centre for Antimicrobial Research and Evaluation (BCARE), Severn Infection Sciences, Pathology Sciences Building, North Bristol NHS Trust, Southmead Hospital, Westbury-on-Trym, Bristol BS10 5NB, UK; Population Health Sciences, University of Bristol, Bristol BS8 2PS, UK; British Society for Antimicrobial Chemotherapy, 53 Regent Place, Birmingham B1 3NJ, UK; Division of Population Health and Genomics, School of Medicine, University of Dundee, Ninewells Hospital and Medical School, Dundee DD1 9SY, UK; Department of Medical Microbiology, Ninewells Hospital and Medical School, Dundee DD1 9SY, UK; Norwich Medical School, University of East Anglia, Norwich NR4 7TJ, UK; AMRHAI (Antimicrobial Resistance and Healthcare Associated Infections), UK Health Security Agency, Colindale, London NW9 5EQ, UK

## Abstract

The British Society for Antimicrobial Chemotherapy Antimicrobial Surveillance Project was established in the United Kingdom (UK) and Ireland in response to dissatisfaction with existing antimicrobial surveillance which, in the 1990s, was fragmented and poorly informative for clinical and public heath purposes. The model developed into an integrated Project that was novel in its management and financing, being a true collaboration between a medical charity, pharmaceutical companies and the laboratories contracted to perform the testing. Separate ‘Programmes’ within an overall ‘Project’ collected and tested community- and hospital-acquired respiratory and bloodstream infection isolates. Cooperation with public sector bodies allowed data collection from the Project to be compared to routinely collected data from National Health Service laboratories. Between 1999 and 2019 the Project delivered test results on almost 100 000 isolates, from 17 bacterial genera to 44 antimicrobial agents. The results were made available annually on a specially constructed web site. In this supplement, we summarize the data collected in a series of papers relevant to the two programmes, discussing the Project’s strengths and deficiencies. Support for the Project declined in the period after 2010, and collection ceased after 20 years, its demise related to changing priorities within the partner organizations. However, the bacterial collection remains and is to be maintained to assist in future work in combating antimicrobial resistance.

## Introduction

The British Society for Antimicrobial Chemotherapy (BSAC) Antimicrobial Surveillance Project, operating in the United Kingdom (UK) and Ireland, sprang out of a dissatisfaction at the then existing mechanisms for assessing the burden of antimicrobial resistance across the British Isles in the late 1990s. At the time, the Public Health Laboratory Service (PHLS, now UKHSA) collected and collated the results of clinical antimicrobial testing in diagnostic microbiology laboratories in areas of England and Wales. Only data for bloodstream isolates were collected and participation in the surveillance was voluntary. Although, by 1998, over 90% of laboratories were providing data on the species isolated from blood cultures, information on antimicrobial susceptibility testing was incomplete.^1^ Systems to measure the prevalence of resistance among key pathogens in Ireland, Northern Ireland and Scotland were even less well established.

The PHLS system was further limited by the capacity of the computer infrastructure both in diagnostic laboratories and centrally, though individual laboratories were able to monitor resistance rates over time.^2^ Computer limitations meant that much data collection was on paper, and consequently participation was highly labour intensive, which limited laboratory participation. An even greater limitation was that no single susceptibility testing method or set of clinical breakpoints was used across the contributing diagnostic laboratories. Most used the modified Stokes Disc Method, which was not validated against MIC testing and had arbitrary definitions of resistance.^3^ A minority, mostly in Scotland and Ireland, used the NCCLS (now CLSI) method. Furthermore, there was no publicly funded system for the routine collection of unusual isolates for phenotypic testing and genotypic exploration of potential resistance mechanisms. Investigation depended on the isolate being sent, at the isolator`s instigation, to the PHLS reference laboratory in Colindale, London, or an academic laboratory.

Pharmaceutical companies were active in resistance surveillance, providing information for antimicrobials relevant to their licenced clinical indications. Such surveillance was often conducted using MIC testing, with occasional mechanistic and genotypic investigations of interesting isolates. However, most studies were conducted at an international level and the number of collecting centres in the British Isles was invariably small and biased towards academic centres and larger laboratories with an interest in antimicrobials. One of the best examples of this type of surveillance was the Alexander Project, which focused on respiratory tract pathogens and was sponsored by SmithKline Beecham, operating internationally for over a decade from 1992.^4^ Multicentre UK specific studies of MIC distributions and mechanisms, such as B lactamase type or aminoglycoside modifying enzymes, were performed *ad hoc*, often supported by industry. These included several surveys of antimicrobial resistance in *Haemophilus influenzae* and *Pseudomonas aeruginosa* both conducted over 15 years^5–8^ or one-off investigations of a particular resistance mechanism of interest.^9^ These surveys typically included 20–25 UK mainland sites, with 1000–5000 isolates collected. Exceptionally, the 1993 *P. aeruginosa* study included Northern Ireland.^8^ In addition, the PHLS reference laboratory undertook MIC-based studies of pneumococci in 1990 and 1995, collecting and characterizing all isolates from around 50 laboratories over a 2-week period each year.^10^

## Design

The BSAC’s response to these limitations involved two major developments—first, development of the BSAC standardized disc susceptibility testing method in 1997–98^3^ and second, the establishment of a BSAC-led antimicrobial surveillance system. The disc method was designed to improve the quality of routine susceptibility testing, supporting both patient treatment and the PHLS’s routine-data-based epidemiological surveillance. The BSAC Surveillance Project, with central collection and testing of isolates, aimed to provide more methodologically robust data when compared with routine collection, and also to allow detailed characterization of unusually resistant isolates.^11^

To deliver the latter objectives, in 1996/7 the BSAC Council formed a ‘Towards a British Surveillance Scheme’ Working Party aimed to enhance the surveillance of antimicrobial resistance in the British Isles and to co-operate with others, notably the then PHLS, to achieve this.^12^ The Working Party, as initially constituted, had six members representing the BSAC (AP MacGowan, Bristol and R Wise, Birmingham), PHLS (DF Brown, Cambridge and DM Livermore, London) and industry (JR Edwards, Zeneca Pharmaceuticals and AR White, SmithKline Beecham). It proposed a pragmatic response to improve surveillance of antimicrobial resistance in the British Isles, based upon:

- An oversight board or group with members of the BSAC and PHLS to manage the process.
- Adoption of the standardized BSAC disc method across PHLS and NHS laboratories to allow feedback of better-quality routine testing results to a central epidemiology unit—putatively the PHLS centre for disease surveillance and control.
- Improved and more demanding external quality assurance of antimicrobial susceptibility testing through UK National External Quality Assessment Service and others.
- Commissioning of central laboratories by the BSAC and its commercial partners to perform MIC-based epidemiological studies on specific pathogen groups collected from sentinel sites. This would allow resistance patterns from the larger routine database from disc test results to be validated using more precise data and molecular analysis of resistance mechanisms.

The BSAC offered support of £30–50 000 per annum to set up and manage the surveillance Project initially and sought further funding from pharmaceutical companies. Whilst the BSAC was taking these significant steps towards a national antimicrobial Resistance Surveillance Project there was increased national attention in this area with the UK Standing Medical Advisory Committee (SMAC) Sub-group on AMR calling for an adequately resourced national surveillance programme.^12,13^

## Development

**Figure 1.**
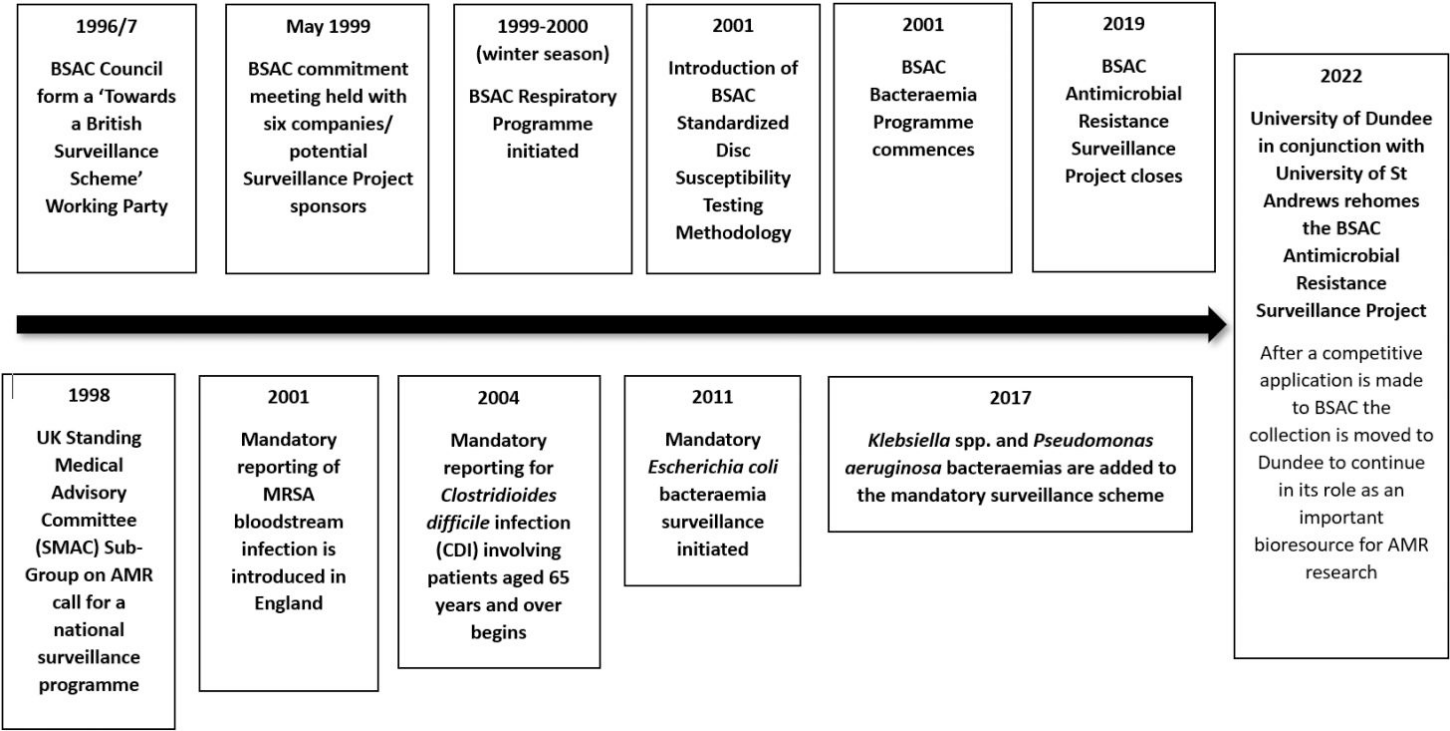
Significant events in the timeline of the BSAC Surveillance Project. BSAC activities above the arrow on the Figure and Governmental Committees or Agencies below the arrow.

In May 1999, the BSAC held a commitment meeting with six companies as potential sponsors of a programme of antimicrobial resistance surveillance in respiratory pathogens to include *Streptococcus pneumoniae, H. influenzae* and *Moraxella catarrhalis*. It was proposed to collect isolates from 20 laboratories across the UK and Ireland and to test 10 antimicrobials. Four sponsors ultimately joined the Programme: Abbott, Aventis, Bayer and SmithKline Beecham. GR Micro in London was selected, by competitive tender, as the central collection and testing laboratory (Lead D Felmingham). The Respiratory Programme began collection and testing of community isolates in the winter of 1999/2000. A Bacteraemia Programme for collection and testing of bloodstream pathogens was added in 2001, initially sponsored by MSD, Pfizer and Wyeth with central testing laboratory at the PHLS Antibiotic Resistance Monitoring and Reference Laboratory in Colindale, London (Lead DM Livermore). The Respiratory Programme was extended in 2008 to include pathogens from hospitalized patients (Lead I Morrisey). A timeline summarizing significant events in the history of the BSAC Surveillance Project is shown on Figure 1.

As planned, the Project was overseen by a Working Party, later Standing Committee, of the BSAC, which was developed to consist of representatives of each sponsor, the PHLS, NHS microbiology and BSAC (Chair AP MacGowan). Management and administrative support were provided to the Standing Committee by a BSAC-appointed co-ordinator, R. Reynolds (1999–2015), V. Martin (2011–16) and C. Horner (2017–20). These individuals had a wide role, working with sponsors, central laboratories, the Society headquarters, Council and others to ensure the smooth running of the Project. They also had a pivotal role in data analysis, presentation and feedback of annual results to sponsors.

## Delivery and Deficiencies

The first published outputs of the Project came in 2001, when data from the Respiratory and Bacteraemia Programmes were presented as posters at the 22nd International Congress of Chemotherapy and 41st Interscience Conference on Antimicrobial Agents and Chemotherapy. A steady flow of further abstracts and papers followed over the next two decades (see https://bsac.org.uk/resistance-surveillance/). From 2002 to 2022, results were accessible on an interactive website offering user-specified tables and graphs of resistance rates and MIC summary measures. This was supported by Micron Research (2002–05) and MRS Web Solutions (2006–22).

A summary of the origins and operation of the Project up to 2007 are given by White^13^ and a list of sponsors and their agents during the whole Project is shown on Table 1. More detail of all antimicrobials tested in the Project is given in Allen *et al.*^17^ In total during the period of the Project, over 100 000 isolates were tested from 1999 to the close of the scheme in 2019 and the papers of this Supplement report results for 99 119 isolates, 17 genera and 44 agents of continuing clinical or surveillance interest.

**Table 1.** Sponsors and agents tested

Sponsor	Antimicrobial Agent
Abbott	ABT-773 (cethromycin) Clarithromycin
Astellas	Cefilavancin (TD-1792) Telavancin
AstraZeneca	Ceftaroline/avibactam Ceftazidime/avibactam Meropenem
Aventis	Levofloxacin Telithromycin
Basilea	BAL30072 BAL30027/meropenem Ceftobiprole Meropenem
Bayer	Amikacin Faropenem Moxifloxacin
Cempra	Azithromycin Solithromycin Telithromycin
Cerexa	Ceftaroline Ceftaroline/avibactam
Cubist	Ceftolozane/tazobactam Daptomycin Tedizolid
GlaxoSmithKline	Gemifloxacin
GeneSoft	Gemifloxacin
Johnson & Johnson	Ceftobiprole Doripenem
Melinta	Delafloxacin
MSD	Ceftolozane/tazobactam Ertapenem Imipenem Imipenem/relebactam Tedizolid Daptomycin
Nabriva	Lefamulin Moxifloxacin
Novartis	Daptomycin Razupenem
Oscient	Gemifloxacin
Pfizer	Ceftaroline Ceftazidime/avibactam Dalbavancin Linezolid
Replidyne	Faropenem
Rib-X	Delafloxacin
Theravance	Cefilavancin (TD-1792)
Wyeth	Minocycline Piperacillin/Tazobactam Tigecycline

Despite the Project’s clear clinical relevance, large scale, quality and the timeliness of the data generated, it was increasingly questioned whether the BSAC should be involved in the routine measurement of antimicrobial resistance, or whether this was rightly a function of Government through agencies such as the UKHSA and its predecessors. In addition, the Society itself was changing and refocusing its priorities away from laboratory-orientated microbiology and towards clinical antimicrobial use, such as antimicrobial stewardship and out-patient parental antimicrobial therapy.^14,15^ These changes in emphasis in the UK and Ireland were played out against the wider international travails of antimicrobial development,^16^ which resulted in fewer sponsors being able or willing to resource antimicrobial resistance surveillance activities in the British Isles. More positively, the quality of routinely collected pathogen susceptibility data had improved since the start of the Project, as bacterial identification became more accurate with the introduction of MALDI-TOF technology and susceptibility tests did so with the wide adoption of BSAC, and then EUCAST, disc testing methodology. Reflecting these changes, the Project—one of the Society’s flagship activities—was terminated in 2019.

In this supplement, which helps to celebrate the 50th anniversary of the first publication of *Journal of Antimicrobial Chemotherapy* in 1975, we summarize and analyse the outputs of the BSAC’s Antimicrobial Resistance Surveillance Project in the period 1999–2019.

Because the Project delivered MIC data, it has been possible to relate its results to current EUCAST breakpoints rather than to contemporaneous definitions of susceptibility and resistance. Moreover, as described in the individual papers in the supplement, mechanisms were characterized—notably including *mecA* expression and ESBL or carbapenemases production. For bloodstream isolates, the strategy of cross-referencing data from the Society’s surveillance programme—with MIC testing of several thousand isolates annually—to the much larger compilation of routine data by the PHLS and its successors, was successful. The lack of a publicly funded surveillance for respiratory pathogens prevented a similar linkage with the Respiratory Programme.

Following this Introduction, Allen *et al.*^17^ provide a review of the methods used to measure the prevalence of resistance in the British Isles over this period and, importantly, critically assess the limitations of the Project’s processes. Next, Reynolds *et al.* report on the resistance burden and trends in Gram-positive and Gram-negative pathogens from patients with bacteraemia^18,19^ and then on the antimicrobial resistance burden and trends in both community and hospital lower respiratory tract pathogens.^20,21^ Horner *et al.*^22^ provides a 20-year overview of the serotypes and resistance rates in *S. pneumoniae* and, finally, Parcell *et al.*^23^ provide a commentary on the lasting legacy of the Project, describing numerous completed and ongoing projects that used, and are using, the Project’s collection of isolates.

Ultimately, the successes of the Project depended on all those involved, and we wish to thank them for their dedication and hard work in delivering a world first in terms of design, funding and execution. Sadly, it is not possible to name everyone involved in such a large and long endeavour, but we remain grateful to all those who lent a hand and supported this work both in the good times, of which there were many, and during the occasional squalls and storms, which inevitably occurred.

We hope that you relish the data presented here and find them useful in your own infection practice. We encourage you to read and assess both the practical approaches to the measurement of antibiotic resistance and the results of such work in a critical but sympathetic way for it is not always easy!

## Future Directions

The isolate collection has been transferred to the University of Dundee, where it remains available to researchers: details are given in Parcell *et al*.^23^

## Transparency declarations

A.P.M. holds research grants/activities with Merck, Shionogi, InfectoPharm, GSK, Roche, BioVersys, VenatoRx Pharmaceuticals, iFAST, Oxford Drug Design, JPIAMR and NIHR; APM provides consultancy advice to Shionogi, Roche, Bicycle Therapeutics and BioVersys. B.J.P. consultancy: Genpax. D.M.L. Advisory Boards or *ad hoc* consultancy: AdjuTec, AstraZeneca, Centauri, GenPax, GSK, Lipovation, Meiji, Paion, Shionogi, Sumitovant, Thermo Fisher and Wockhardt; Paid lectures—bioMérieux, Pfizer, Shionogi and Zuellig Pharma. Relevant shareholdings or options—GenPax, GSK, Merck and Revvity amounting to less than 10% of portfolio value. DML also has shareholdings in Arecor, Celadon Pharma, Destiny Pharma, Genincode, Probiotix, Rua Life Sciences, SkinBiotherapeutics and VericiDx (all with research/products pertinent to medicines or diagnostics) through Enterprise Investment Schemes, but has no authority to trade these holdings directly. R.R. has nothing to declare.
